# Notes for Practitioners

**Published:** 1888-01-21

**Authors:** Geo. W. Potter


### Notes for Practitioners.

By G. W. Potter, M.D.

A Safe and Sure Hypnotic is always a welcome addition to the practitioner's resources. *Amylene hydrate* is the last new candidate for favour of this kind. Wartz was the original discoverer; and Professor V. Mehring and Dr. Scharschmidt have experimented pretty freely both on men and animals. So far as the experimenters report, the results may be considered decidedly favourable The administration of sufficient doses of the drug procured sound sleep in the majority of cases. The sleep lasted six or seven hours, and it is said that no ill effects followed—no nausea, no headache, no digestive disturbance. The dose required varied from 20 to 80 grains. A good vehicle for administration is a mixture made with a little red wine sweetened with sugar. So far as appears at present, there is not much risk attending the use of the drug, its physiological action appearing to be exerted first on the cerebrum, and not markedly on the cerebellum, unless the dose be large. Experiments on dogs showed that deep sleep was produced in from ten minutes to half-an-hour, and that this sleep was prolonged, without any accompanying alarming effects on the heart and respiration. An increase of the dose acted powerfully on the medulla, bringing the vagus into strong inhibitory action. Respiration became slower, and death resulted from arrest of the heart. In dogs, after the administration of a sufficient and safe quantity, the respiratory movements fell from twenty to sixteen, a fall similar to that which occurs in normal sleep, and the differences in pulse rate and blood pressure were hardly perceptible. *Amylene hydrate* is described as a clear, colourless, slightly oily liquid, which floats upon water, and has a specific gravity of 0·81. It boils a little above the boiling point of water—102·5 C. It has an odour resembling that of paraldehyde, and very slightly that of camphor. The taste is that of a hot aromatic, and the after-effects are somewhat pungent. It is freely soluble in alcohol, but only slightly so in water. Chemically, it appears to be a tertiary amyl-alcohol, and in constitution is said to be dimethylethyl carbinol, $\left.\begin{matrix}(CH_3)2\\ C_2H_4\end{matrix}\right\}C.OH.$ Those who have had experience in hypnotics will not hastily form a judgment as to the value of the new aspirant for a place in the medical armamentarium; but there appears to be no reason whatever why it should not have an extended trial forthwith. It is desirable that all practitioners should lend a helping hand in the establishment of the reputation of every new drug.

---

The Advantages of Early Surgical Interference in certain instances were well shown in a case reported at the Medical and Chirurgical Society of London by Dr. W. B. Cheadle, F.R.C.P., and Mr. Thomas Smith, F.R.C.S. A girl aged nine had swallowed a metal pencil cap. The cap stuck in the throat, and was forced down by a probang, apparently into the stomach. There was great pain at the time, and violent coughing. Four days later, dulness on percussion and imperfect entry of air were noted on the left side of the chest. This continued, and eleven days after the accident the whole of the left side was dull, and no respiratory murmur could be heard. The stomach was displaced upwards to the nipple line, and the left half of the thorax was retracted, indicating almost complete collapse of the lung. At this period the respirations were 30 and the pulse 92; but the temperature was below the normal—97·8 deg. There was no difficulty of breathing, but an occasional short cough. It was judged that the pencil cap, which was about an inch long and a quarter of an inch in diameter, had lodged in the extreme end of the left bronchus. It was decided, after consultation, that tracheotomy offered the most reasonable solution of the difficulty, and that it had better be performed before the case should become complicated by inflammatory changes. The isthmus of the thyroid was divided between two ligatures, the trachea freely opened, and the edges of the tracheal wound attached to the margins of the skin by silk sutures. A long probe was then passed into the bronchus, and the foreign body was discovered in exactly the place where it had previously been judged to be lodged. The open end of the cap was outwards. A pair of curved forceps, with external grip, which had previously been tried on a facsimile of the cap and found to hold firmly, were then introduced, and the dangerous guest was dislodged without difficulty. The object of attaching the tracheal edge of the wound to the skin was to secure a clear opening, and the plan was found to answer excellently. The patient made a rapid and uninterrupted recovery, except that she was slightly feverish for a short time the day after the operation. If the cap had not been removed at all, the patient must have died a lingering and painful death; if it had been left until suffocation was imminent, and then removed, the chances of recovery would have been diminished a hundredfold. The moral of the story is, that if an operation is inevitable, it is generally best to perform it with as little delay as possible.

---

Acute Traumatic Tetanus is a formidable enemy, and often greatly taxes the resources of the medical art for its successful treatment. Mr. Butlin reports a case in which salicin, with bromide of potassium, was apparently the effective agent in checking the disease and giving the start towards recovery. A young man, aged 26, of rather intemperate habits, cut his arm severely in June last, by thrusting his hand through a window. The wound appeared to make good progress for a fortnight, but on July 15th he complained of pain in the epigastrium. In the course of the day his face began to stiffen, and there were twitches of the muscles of the face and the backs of the legs. These grew worse until the 19th. At this time the "risus sardonicus" was well marked, and spasm was elicited when the patient was sharply or loudly spoken to. He was placed in a warm room, fitted with carpets and curtains to deaden sound, and full doses of chloral hydrate and bromide of potassium were administered. But he grew worse: the breathing became laboured, and the pulse was rapid. On the 22nd he appeared to be actually dying; the respirations and pulse could not be counted, and spasms occurred with extreme rapidity. At one p.m. it was resolved to try the effect of salicin, instead of chloral, with the bromide. A twenty-grain dose, with the same quantity of bromide, was given. The dose was repeated every two hours. At five p.m. (four hours after the first dose) the frequency of the spasms had much diminished; they occurred only once in ten minutes; the pulse could be easily counted, and numbered 120; it was much stronger. As had been anticipated, profuse perspirations set in. On the following morning the intervals between the spasms had increased to twenty minutes; the breathing was easy, and the pulse slower and stronger. The dose was now ordered to be given every four hours. The patient gradually improved; but it was several days before the pain in the epigastrium ceased. At the end of six weeks convalescence was fairly established, and the case progressed to recovery. The probability is that the profuse perspiration marked the crisis of the attack, and that the recovery was due to the full doses of salicin. It may be hoped that this case will be remembered, and that other experiments will be made on the same lines. The usual depressant effects of salicin on the heart's action appears to have been absent in this instance. It is remarkable that in spite of the full and frequently repeated doses the pulse became rapidly slower and *stronger*. A case of this kind points to the possibility that salicin may prove to be of service in delirium tremens. It is well worth a trial.

